# A bifunctional aminoglycoside acetyltransferase/phosphotransferase conferring tobramycin resistance provides an efficient selectable marker for plastid transformation

**DOI:** 10.1007/s11103-016-0560-x

**Published:** 2016-11-17

**Authors:** Iman Tabatabaei, Stephanie Ruf, Ralph Bock

**Affiliations:** Max-Planck-Institut für Molekulare Pflanzenphysiologie, Am Mühlenberg 1, 14476 Potsdam-Golm, Germany

**Keywords:** Plastid transformation, *Nicotiana tabacum*, Selectable marker, Tobramycin, Bifunctional enzyme

## Abstract

**Key message:**

A new selectable marker gene for stable transformation of the plastid genome was developed that is similarly efficient as the *aadA*, and produces no background of spontaneous resistance mutants.

**Abstract:**

More than 25 years after its development for *Chlamydomonas* and tobacco, the transformation of the chloroplast genome still represents a challenging technology that is available only in a handful of species. The vast majority of chloroplast transformation experiments conducted thus far have relied on a single selectable marker gene, the spectinomycin resistance gene *aadA*. Although a few alternative markers have been reported, the *aadA* has remained unrivalled in efficiency and is, therefore, nearly exclusively used. The development of new marker genes for plastid transformation is of crucial importance to all efforts towards extending the species range of the technology as well as to those applications in basic research, biotechnology and synthetic biology that involve the multistep engineering of plastid genomes. Here, we have tested a bifunctional resistance gene for its suitability as a selectable marker for chloroplast transformation. The bacterial enzyme aminoglycoside acetyltransferase(6′)-Ie/aminoglycoside phosphotransferase(2″)-Ia possesses an N-terminal acetyltransferase domain and a C-terminal phosphotransferase domain that can act synergistically and detoxify aminoglycoside antibiotics highly efficiently. We report that, in combination with selection for resistance to the aminoglycoside tobramycin, the *aac(6*′*)-Ie*/*aph(2*″*)-Ia* gene represents an efficient marker for plastid transformation in that it produces similar numbers of transplastomic lines as the spectinomycin resistance gene *aadA*. Importantly, no spontaneous antibiotic resistance mutants appear under tobramycin selection.

**Electronic supplementary material:**

The online version of this article (doi:10.1007/s11103-016-0560-x) contains supplementary material, which is available to authorized users.

## Introduction

The development of technologies to engineer plastid (chloroplast) genomes (Boynton et al. 1988; Svab et al. 1990) has opened up new exciting opportunities to study virtually all aspects of plastid biology using in vivo approaches. Over the last three decades, application of the technology has provided novel insights into plastid gene expression (e.g., Kuras and Wollman 1994; Staub and Maliga 1993; Hajdukiewicz et al. 1997; Bock and Koop 1997), chloroplast gene functions (e.g., Monod et al. 1994; Ruf et al. 1997; Hager et al. 1999), plastid inheritance (Ruf et al. 2007; Svab and Maliga 2007) and genome evolution (Huang et al. 2003; Stegemann et al. 2003, 2012). Moreover, plastid transformation technologies have also stirred considerable excitement among plant biotechnologists, because transgene expression from the plastid genome offers a number of unique attractions, such as foreign protein expression to very high levels (De Cosa et al. 2001; Oey et al. 2009a), convenient stacking of multiple transgenes in synthetic operons (Lu et al. 2013) and improved transgene containment due to the maternal mode of plastid inheritance in most crops which largely prevents unwanted transgene transmission via pollen (reviewed, e.g., in Maliga 2004; Bock 2015). In recent years, a large number of proof-of-concept studies have demonstrated the great potential of the transplastomic technology in molecular farming (Staub et al. 2000; Tregoning et al. 2003; Oey et al. 2009b), metabolic engineering (Apel and Bock 2009; Bohmert-Tatarev et al. 2011; Fuentes et al. 2016) and resistance engineering (De Cosa et al. 2001; Ye et al. 2001; Zhang et al. 2015).

A major technical challenge still lies in the extension of the plastid transformation technology to new species and, especially, to major food crops (Bock 2014). Currently, plastid engineering is restricted to a handful of species, with the unicellular alga *Chlamydomonas rheinhardtii* and the seed plant model tobacco (*Nicotiana tabacum*) being the only species where the technology is routine in at least a number of laboratories. In addition to limitations related to the available tissue culture systems and plant regeneration protocols, the paucity of selectable marker genes for plastid transformation and the complete lack of suitable selection markers for certain groups of species represent the most serious obstacle to the development of workable transformation protocols for additional species. For example, cereals, the world’s most important food crops, are recalcitrant to chloroplast transformation, because they are naturally resistant to spectinomycin (Fromm et al. 1987), the most commonly used antibiotic for the selection of transplastomic cells. The lack of a suitable selectable marker gene is also the main obstacle to the development of a mitochondrial transformation technology in plants (Li et al. 2011).

The nearly universally employed selectable marker gene for plastid transformation is the *aadA* gene (Goldschmidt-Clermont 1991; Svab and Maliga 1993). It was identified in a strain of the gut bacterium *Escherichia coli* and encodes an aminoglycoside 3″-adenylyltransferase. This enzyme covalently modifies the aminoglycoside antibiotics spectinomycin and streptomycin by attaching an AMP residue to the antibiotic molecules. Unlike the unmodified antibiotics, the adenylylated drugs do not bind to the 30S subunit of the prokaryotic 70S ribosomes of the chloroplast and, therefore, do not block plastid protein biosynthesis. The identification of spectinomycin as selection agent for transplastomic cells in conjunction with the *aadA* marker (Goldschmidt-Clermont 1991; Svab and Maliga 1993) was a lucky strike. Despite great efforts to develop alternative markers, the *aadA* gene has remained unparalleled in its efficiency. This is likely due to the high enzymatic activity of the AadA protein and the high specificity of spectinomycin as a potent inhibitor of plastid translation. A few alternative selectable markers have been developed for tobacco plastid transformation, including the *nptII* gene encoding a neomycin phosphotransferase that confers resistance to kanamycin (Carrer et al. 1993), the *aphA-6* gene that encodes an aminoglycoside phosphotransferase also conferring kanamycin resistance (Huang et al. 2002), and the *cat* gene encoding chloramphenicol acetyltransferase and conferring resistance to chloramphenicol (Li et al. 2011). However, due to their substantially lower efficiency than *aadA*-based selection, they have not become widely adopted. It is also interesting to note that there are a number of markers that do not work for the primary selection of transplastomic cell lines, even though they confer good secondary resistance to the corresponding selection agents after their introduction into the plastid genome via selection for the *aadA* marker. These secondary markers include, for example, herbicide resistances (Ye et al. 2003) and resistances to toxic d-amino acids (Gisby et al. 2012). The reasons for these markers not being suitable for primary selection of transplastomic cells are not entirely clear, although lethality of the corresponding selection agents has been suggested as a possible explanation (Ye et al. 2003).

In summary, although the *aadA* gene provides a highly efficient and specific selectable marker, there is a need to develop alternative markers for plastid transformation to (a) extend the species range of the technology, and (b) facilitate the multistep engineering of plastid genomes, for example, by sequential introduction of multiple transgenes (supertransformation).

**Fig. 1:**
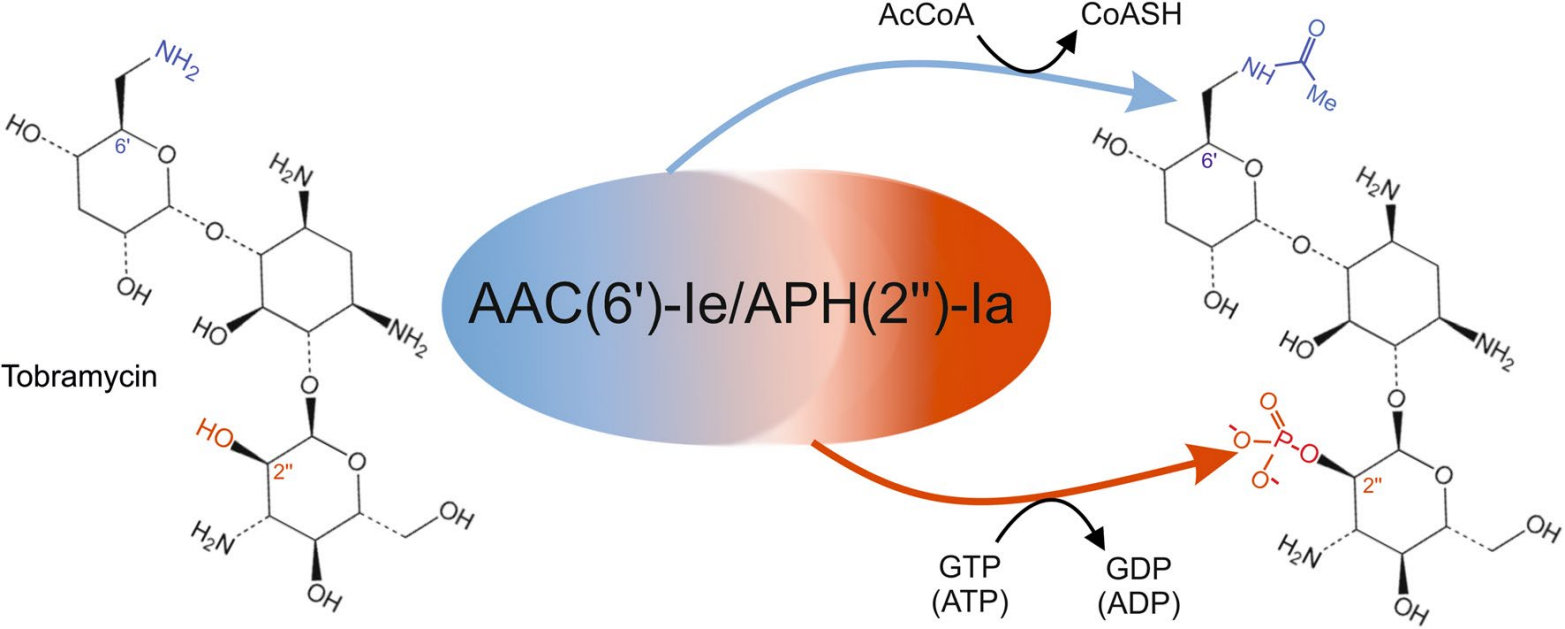
The bifunctional AAC(6′)-Ie/APH(2″)-Ia enzyme has two active domains. The AAC(6′) domain catalyzes an acetylation reaction, using acetyl-CoA as the acetyl donor, to the 6′-amino group of ring I of aminoglycoside antibiotics. Exemplarily, the structure of tobramycin is shown here. The APH(2″) domain uses GTP (or ATP) as phosphate donor and phosphorylates the 2″-hydroxyl group of ring III of the aminoglycoside molecule (Frase et al. 2012)

Aminoglycosides are a class of broad-spectrum antibiotics that inhibit prokaryotic translation through high-affinity binding to the small (30S) subunit of the 70S ribosome (Tenson and Mankin 2006). Bacteria can acquire resistance to aminoglycosides by enzymatic modification of the antibiotic molecules. Resistance-conferring, aminoglycoside-modifying enzymes are biochemically classified into (a) aminoglycoside *O*-nucleotidyltransferases (e.g., the AadA), (b) aminoglycoside *O*-phosphotransferases, and (3) aminoglycoside *N*-acetyltransferases (Mingeot-Leclercq et al. 1999). During evolution and, presumably, under selective pressure from antibiotics present in the environment, a few bifunctional enzymes have arisen that harbor two aminoglycoside-modifying activities. These bifunctional enzymes are likely the result of gene fusion events and are thought to detoxify their host cells more efficiently than the monofunctional enzymes they evolved from. The bifunctional enzyme aminoglycoside acetyltransferase(6′)-Ie/aminoglycoside phosphotransferase(2″)-Ia, AAC(6′)-Ie/APH(2″)-Ia, is responsible for high-level antibiotic resistance in Gram-positive bacteria, including pathogenic strains of *Enterococcus* and *Staphylococcus* (Frase et al. 2012). The enzyme comprises an N-terminal AAC(6′) domain (acetyltransferase domain) and a C-terminal APH(2″) domain (phosphotransferase domain; Fig. 1) that can function independently of each other. By phosphorylation and/or acetylation, the AAC(6′)-Ie/APH(2″)-Ia enzyme (for brevity, subsequently referred to as AAC6-APH2) inactivates a broad range of aminoglycoside antibiotics. 4,6-disubstituted and so-called atypical aminoglycosides are particularly good substrates of its phosphorylation activity (Frase et al. 2012). 4,6-disubstituted aminoglycosides include, for example, kanamycin, tobramycin, amikacin, gentamicin C and sisomicin, whereas the 6-unsubstituted antibiotic neamine represents an atypical aminoglycoside.

Due to their bacterial origin, chloroplasts possess a prokaryotic translational apparatus that relies on 70S ribosomes (Tiller and Bock 2014). Consequently, protein biosynthesis in chloroplasts displays similar antibiotic sensitivities as bacterial translation (Tenson and Mankin 2006; Bock 2015). Here, we have explored the possibility to use the gene for the bifunctional aminoglycoside-modifying enzyme AAC6-APH2 as a new selectable marker for plastid transformation in seed plants. We show that transplastomic lines can be obtained by selection for either tobramycin or gentamicin C (gentamicin). Importantly, in combination with tobramycin selection, the *aac6-aph2* gene produces comparable numbers of transplastomic lines as the *aadA* marker, while giving no background of spontaneous antibiotic-resistant mutants.

## Materials and methods

### Plant material and growth conditions

Tobacco plants (*Nicotiana tabacum* cv. Petit Havana) were raised from seeds under aseptic conditions on agar-solidified (MS) medium (Murashige and Skoog 1962) containing 30 g/L sucrose. For biolistic transformation, young leaves were harvested from 4-week-old plants. Regenerated transplastomic shoots were rooted and propagated on MS medium with 30 g/L sucrose and 30 mg/L tobramycin sulfate (Duchefa or Sigma) or gentamicin sulfate (Duchefa). After rooting, homoplasmic plants were transferred to soil and grown under standard greenhouse conditions. To test for homoplasmy and maternal inheritance, seeds from appropriate crosses were germinated on MS medium containing 50 mg/L tobramycin sulfate or gentamicin sulfate.

### Vector construction

**Fig. 2:**
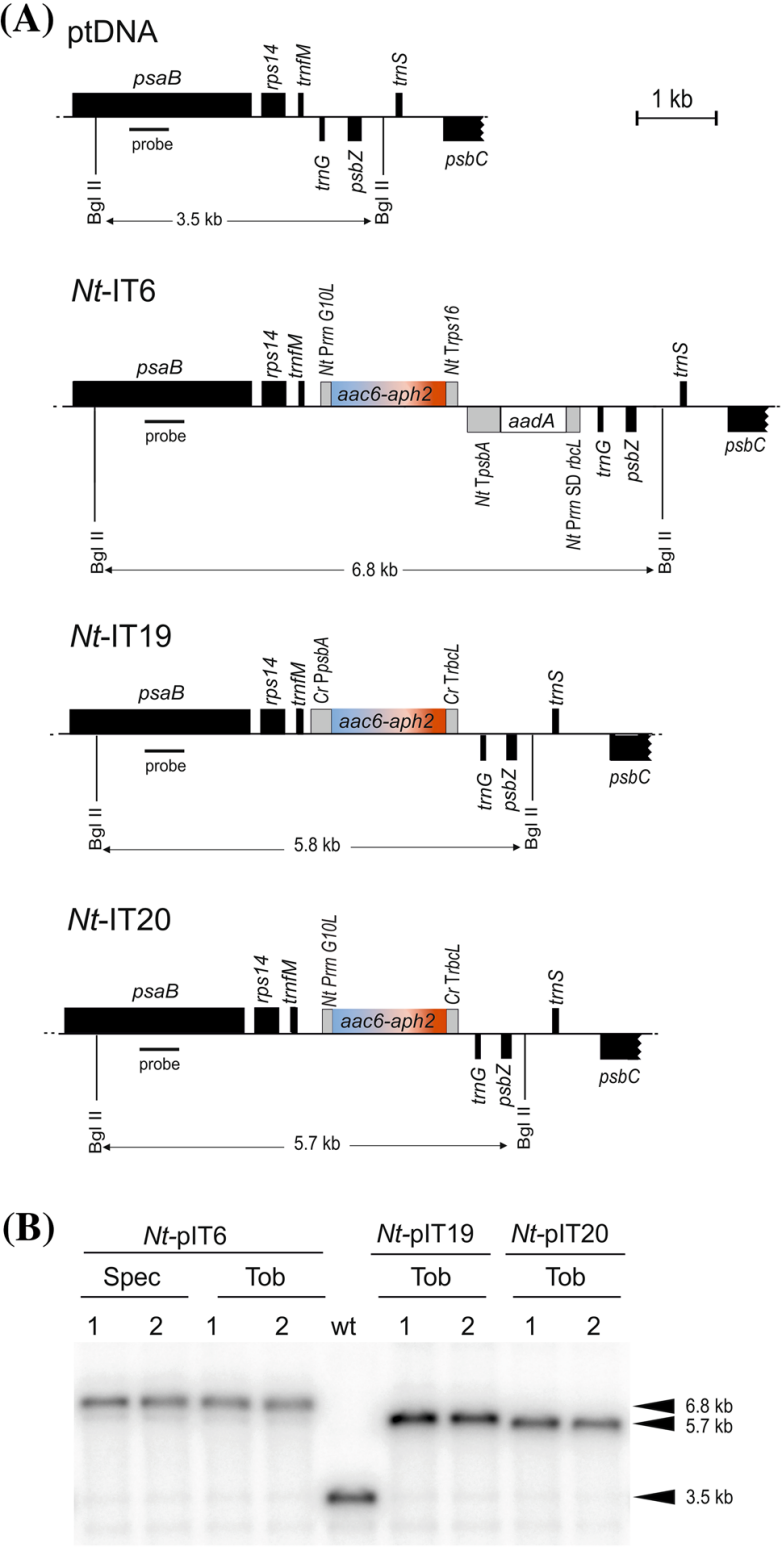
Generation of transplastomic tobacco plants by tobramycin selection. **a** Physical maps of the targeting region in the wild-type chloroplast genome (ptDNA) and the genomes of the transplastomic lines (*Nt*-IT) obtained with plastid transformation vectors pIT6, pIT19 and pIT20. *Filled black boxes* represent tobacco plastid genes, the *aadA* marker present in vector pIT6 is shown as an *open box* and the new *aac6-aph2* selectable marker (present in all pIT vectors) as a *blue*-*red box*. Expression elements (promoters, 5′ UTRs and 3′ UTRs) are represented as *grey boxes* and denoted by the source organism (*Nt: Nicotiana tabacum; Cr: Chlamydomonas reinhardtii*) and the source gene (P*rrn*: rRNA operon promoter; *G10L*: Shine-Dalgarno sequence from the bacteriophage T7 *gene 10*; T*rps16*: 3′ UTR from the plastid *rps16* gene; SD *rbcL*: Shine-Dalgarno sequence from the plastid *rbcL* gene; T*psbA*: 3′ UTR from the plastid *psbA* gene; P*psbA*: promoter from the *psbA* gene; T*rbcL*: 3′ UTR from the *rbcL* gene). Genes *above the lines* are transcribed from *left to right*, genes *below the lines* are transcribed in the *opposite direction*. A PCR product derived from the tobacco *psaB* gene was used as probe in restriction fragment length polymorphism (RFLP) analyses and is represented as a *black bar*. The expected sizes of plastid DNA fragments in RFLP analyses with the restriction enzyme BglII are indicated *below each map*. **b** RFLP analysis of transplastomic lines. DNA samples of the wild type (wt) and several independently generated antibiotic-resistant lines obtained from primary selection or the first regeneration round were digested with BglII, separated in 1% agarose gels, blotted and hybridized to the radiolabeled *psaB* probe shown in *panel* (**a**). The probe detects the expected 3.5 kb fragment in the wild type, a 6.8 kb fragment in the transplastomic lines obtained with vector pIT6, and a 5.8 and 5.7 kb fragment in the transplastomic lines produced with vectors pIT19 and pIT20, respectively

pIT6 is a dual selectable chloroplast transformation vector that was constructed based on plastid transformation vector pKP9 (Zhou et al. 2008). The coding region of the bifunctional resistance gene *aac(6*′*)-Ie*/*aph(2*″)*-Ia* (subsequently abbreviated *aac6-aph2*; Frase et al. 2012) was codon optimized for the tobacco plastid genome and chemically synthesized (GeneArt, Regensburg, Germany) with the start codon being part of an NcoI restriction site and the stop codon followed by an XbaI site. The coding region was then subcloned as an NcoI/XbaI fragment into a similarly cut chloroplast expression cassette consisting of the ribosomal RNA operon promoter (*Nt* P*rrn*), the T7 *gene 10* leader sequence (*G10L*, followed by the translation initiation codon as part of an NcoI restriction site; Zhou et al. 2008) and the 3′ UTR from the chloroplast *rps16* gene (*Nt* T*rps16*; Wurbs et al. 2007). The chimeric *aac6-aph2* gene cassette was then cloned as a SacI/HindIII restriction fragment into vector pKP9, which contains the spectinomycin resistance gene *aadA* (Svab and Maliga 1993) as an additional selectable marker gene (Fig. 2; Table S1).

To be able to conduct transformation experiments with vectors containing only the *aac6-aph2* gene cassette as selectable marker and to avoid flip-flop recombination between the two *Nt* P*rrn* promoter copies present in pIT6 (Rogalski et al. 2006), chloroplast transformation vectors pIT19 and pIT20 were constructed. In pIT19, the *aac6-aph2* gene is driven by the *psbA* gene promoter (P*psbA*) from *Chlamydomonas reinhardtii* (*Cr* P*psbA*; Fleischmann et al. 2011; Table S1). In pIT20, the *aac6-aph2* gene is under the control of the tobacco ribosomal RNA operon promoter (*Nt* P*rrn*). In both vectors, the 3′ UTR from the *C. reinhardtii* plastid *rbcL* gene (*Cr* T*rbcL*; Zou et al. 2003) was used as terminator for the marker gene cassette (Fig. 2). pIT19 was constructed by PCR amplification of the *aac6-aph2* coding region, followed by cloning as a NcoI/SphI restriction fragment into the identically cut plasmid pDK305. pDK305 is a derivative of plastid transformation vector pRB95 (Ruf et al. 2001) and contains a chimeric *aadA* gene driven by the *Cr* P*psbA* promoter and the *Cr* T*rbcL* terminator. For construction of pIT20, the *aac6-aph2* gene together with the ribosomal RNA operon promoter (*Nt* P*rrn*) and the T7 *gene 10* leader sequence was amplified and cloned as XhoI/SphI restriction fragment into the XhoI/SphI-digested plasmid pIT19. The DNA sequences of all coding regions and expression elements (promoters, 5′ UTRs and 3′ UTRs) used in this study are given in Table S1.

### Plastid transformation and selection of homoplasmic transplastomic lines

For chloroplast transformation, young leaves harvested from aseptically grown tobacco plants were biolistically bombarded with plasmid DNA (pIT6, pIT19, pIT20 and pDK305) coated onto 0.6 µm gold particles (BioRad) using the DuPont PDS-1000/He biolistic gun with the hepta adaptor setup. After bombardment, the leaves were cut into small pieces (~5 × 5 mm in size) which were then placed onto the surface of an MS-based selective regeneration medium containing tobramycin sulfate (30, 40 or 50 mg/L) or gentamicin sulfate (50 mg/L). Spectinomycin-resistant shoots were selected on medium with 500 mg/L spectinomycin (Svab and Maliga 1993). Selection was conducted under 25 µE m^−2^ s^−1^ light intensity in a 16 h light/8 h dark cycle. When a medium change was performed, the leaf pieces were transferred to fresh selection medium after 3–4 weeks. Transplastomic lines selected on spectinomycin medium were confirmed by an additional regeneration round on medium containing 500 mg/L streptomycin (Bock 2001). Primary resistant lines in the spectinomycin selection system typically appeared after 4–10 weeks, primary tobramycin or gentamicin-resistant lines appeared after 10–18 weeks. Antibiotic-resistant calli or leaf pieces from regenerating shoots were transferred to fresh selection medium for further propagation and purification of homoplasmic transplastomic tissue.

For quantification of transformation efficiencies, primary resistant lines and confirmed transplastomic lines were counted (Table 1). Transformation frequencies were expressed as number of transformants divided by the number of selected explants. It should be noted that, in plastid transformation, there can be substantial variation in transformation efficiency between experiments. The source(s) of this variation are currently not fully understood, but it is believed that a number of factors contribute, including the physiological status of the bombarded leaf material, the quality of the gold particle preparation and subtle variations in the many parameters involved in tissue culture and selection.

**Table 1:** Statistics of the chloroplast transformation experiments with *aac6-aph2*-containing vectors and of the control experiments with *aadA*-containing vectors

Vector	Selection agent (mg/L)	Number of shots^b^	Selected explants	Medium change	Primary resistant lines	Resistant in additional regeneration	Confirmed by RFLP	Efficiency of selection (%)^d^	Trans-formation efficiency^f^	Appearance of events (weeks)	Homoplasmic in primary selection
pIT6	Gent 50	2	700	No	41	7	7	7	0.01	12–14	0
pIT19	Gent 50	2	679	No	20	0	–	–	0	12–14	–
pIT6	Siso 25	2	770	All	5	0	–	–	0	8–12	–
pIT19	Siso 25	2	672	No	21	0	–	–	0	8–12	–
pIT6	Siso 35	2	735	All	3	0	–	–	0	8–12	–
pIT6	Tob 30	2	700	50%	62	29	29	46	0.041	8–12	0
pIT6	Tob 40	2	700	50%	43 + 15^c^	21 + 12	21 + 12	48/80^e^	0.047	8–12	0
pIT19	Tob 50	2	728	No	36	27	27	75	0.037	12–18	6
pIT20	Tob 50	2	686	No	157	60	60	39	0.087	10–32	26
pIT20	Tob 50	2	721	50%	37 + 28	28 + 26	28 + 26	75/92^e^	0.074	13–23	3
pIT20	Tob 75	2	749	50%	0	–	–	–	0	–	–
pIT20	Tob 100	2	749	50%	0	–	–	–	0	–	–
pDK305	Spec 500	2	700	No	97	63^a^	–	64	0.09	5–15	–
pIT6	Spec 500	2	840	No	30	23^a^	23	76	0.027^g^	4–10	–

*Tob* tobramycin, *Gent* gentamicin, *Spec* spectinomycin

–Not performed

^a^Tested on streptomycin-containing medium

^b^Using the hepta adaptor and bombarding a Petri dish fully covered with leaves

^c^Number without medium change + number with medium change

^d^Number of confirmed transformants divided by the number of primary resistant lines

^e^Number without medium change/number with medium change

^f^Number of transformants divided by the number of selected explants

^g^Probably an underestimate (selection was discontinued after 10 weeks)

### Crosses and inheritance assays

To confirm the homoplasmic state of transplastomic lines and maternal transgene inheritance, plants were grown to maturity under standard greenhouse conditions. Upon flowering, plants were either self-pollinated or reciprocally crossed to wild-type plants. Seeds were harvested and assayed by germination on tobramycin-containing (50 mg/L tobramycin sulfate) or gentamicin containing MS medium (50 mg/L gentamicin sulfate).

### Isolation of nucleic acids and DNA gel blot analyses

Total genomic DNA was isolated from fresh leaf material using a cetyltrimethylammoniumbromide (CTAB)-based protocol (Doyle and Doyle 1990).

For Southern blot analyses, DNA samples were digested with the restriction enzyme BglII, separated by gel electrophoresis in 1% (w/v) agarose gels and transferred onto Hybond XL nylon membranes (GE Healthcare) by capillary blotting using a standard protocol. As RFLP probe, a 550 bp PCR amplicon derived from the *psaB* coding region (Fig. 2a) was used (Wurbs et al. 2007). The amplified fragment was purified by agarose gel electrophoresis and extraction from the excised gel slice using the GFX PCR (DNA and Gel Band Purification) kit. Radiolabelling was performed with [α^32^P]dCTP by random priming (Multiprime DNA labeling system, GE Healthcare) according to the protocol of the supplier. Hybridizations were performed overnight at 65 °C. Following standard washing steps, the membranes were exposed to autoradiographic screens and then scanned in a Typhoon TRIO + scanner (GE Healthcare).

## Results

Construction of plastid transformation vectors based on the bifunctional *aac6-aph2* resistance marker and identification of suitable selection conditions for chloroplast transformation.

To explore the possibility to use the bifunctional *aac6-aph2* gene as a selectable marker for chloroplast transformation, we first tested the sensitivities of tobacco leaf explants to those aminoglycosides that are efficiently detoxified by the AAC6-APH2 enzyme in bacteria (Frase et al. 2012). The following antibiotics were included in these assays: G418 (also known as geneticin), gentamicin, tobramycin, sisomicin, kanamycin B and kanamycin A. Tobacco cells turned out to be sensitive to all of these drugs, albeit the minimum antibiotic concentration required to fully suppress plant regeneration differed between the tested aminoglycosides (Fig. S1). For example, while G418 applied at a concentration of 5 mg/L was sufficient to cause rapid bleaching of the leaf explants and inhibit shoot regeneration, tobramycin needed to be applied at an approximately tenfold higher concentration to achieve complete suppression of callus growth and regeneration (Fig. S1). However, previous work on selectable marker development for plastids has revealed that high antibiotic sensitivity is not necessarily correlated to the efficiency of selection for transplastomic clones. For example, in the currently most efficient transplastomic selection system (based on spectionomycin resistance conferred by chimeric *aadA* genes), typically antibiotic concentrations of as much as 500 mg/L are used to prevent the occasional regeneration of non-resistant plants (escapees). In fact, nonlethal selection has been proposed to be advantageous in plastid transformation in that it allows the transformed cells exposed to the selection agent to survive for a sufficiently long time to establish the antibiotic resistance (Ye et al. 2003). Based on these considerations, we chose tobramycin, sisomicin and gentamicin as selection agents to be tested in chloroplast transformation experiments.

To be able to conduct chloroplast transformation experiments with the bifunctional *aac6-aph2* gene as selectable marker, a set of plastid transformation vectors was constructed (see “Materials and methods”; Fig. 2). Vector pIT6 was built to test whether the *aac6-aph2* gene is capable of conferring resistance upon expression from the plastid genome. It contains the *aadA* marker in addition to the *aac6-aph2* gene and, therefore, allows for introduction of the *aac6-aph2* gene into the chloroplast genome by standard spectinomycin selection. In addition, the use of this vector facilitates side-by-side comparison between the standard *aadA*-based spectinomycin selection of transplastomic lines (Svab and Maliga 1993) and any new selection scheme potentially based on *aac6-aph2*. By contrast, vectors pIT19 and pIT20 were designed for primary selection for AAC6-APH2-based antibiotic resistances (Fig. 2a). They contain the *aac6-aph2* gene as the sole selectable marker gene and only differ in the expression signals driving *aac6-aph2* expression. While, in vector pIT20, the *aac6-aph2* is under the control of the strongest known expression signals for tobacco chloroplasts (a fusion of the ribosomal RNA operon promoter to a synthetic Shine-Dalgarno sequence derived from the bacteriophage T7 *gene 10* leader sequence; Kuroda and Maliga 2001; Oey et al. 2009a), in vector pIT19, the marker gene is driven by a considerably weaker promoter and 5′ UTR (derived from the *psbA* gene of the unicellular green alga *Chlamydomonas reinhardtii*; Emadpour et al. 2015).

### Plastid transformation using *aac6-aph2* as selectable marker gene

Chloroplast transformation experiments were conducted with all three vectors using the biolistic protocol (Svab et al. 1990; Svab and Maliga 1993) and tobramycin, sisomicin and gentamicin as selection agents (Table 1). To allow for comparison of selection efficiencies, part of the samples bombarded with the *aadA*-containing vector pIT6 were subjected to selection for spectinomycin resistance. As a control, a standard *aadA*-based plastid transformation vector (pDK305; see “Materials and methods”; Ruf et al. 2001) was also included in the experiments.

**Fig. 3:**
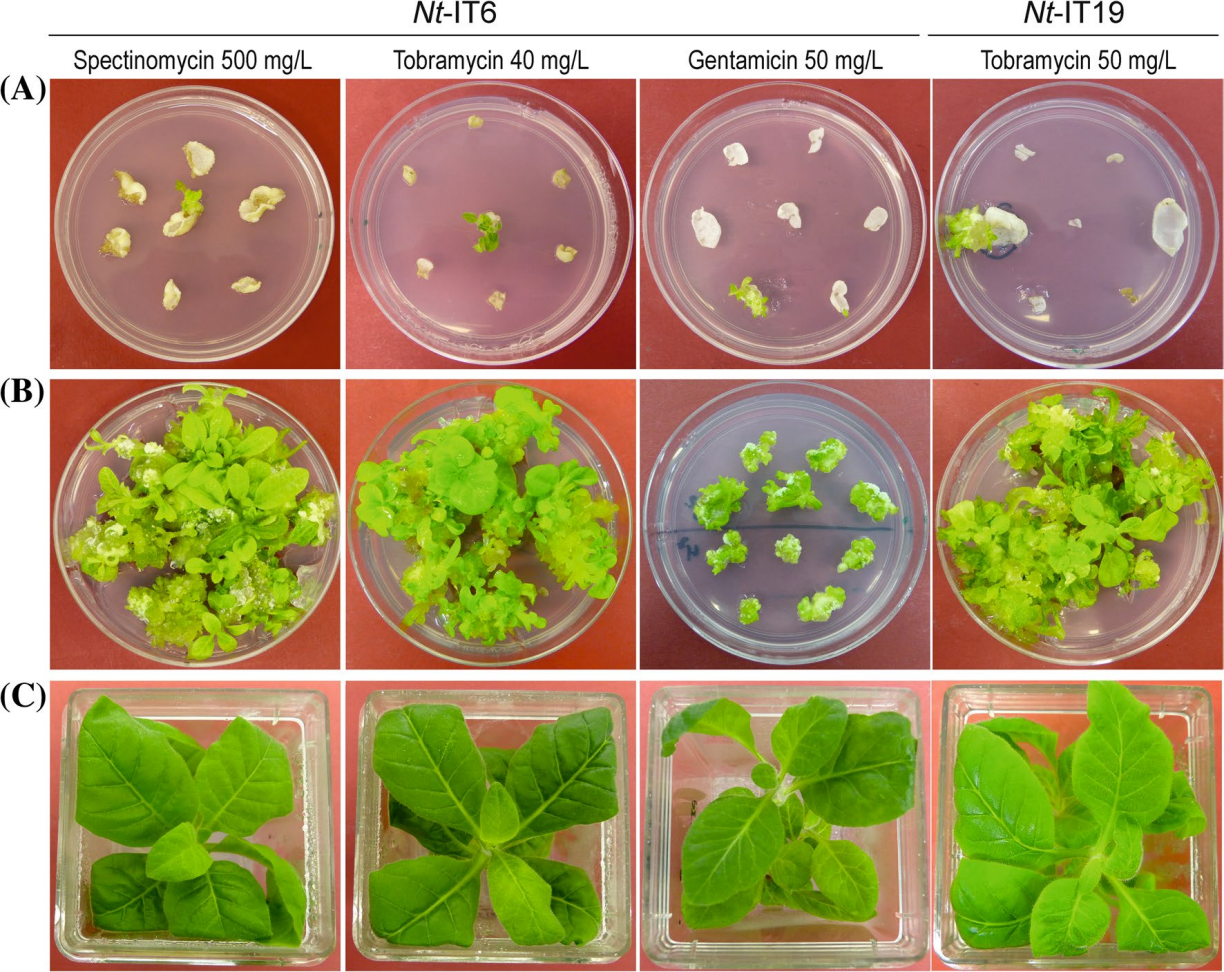
Generation of transplastomic tobacco lines by selection for tobramycin resistance or gentamicin resistance. **a** Primary selection of transplastomic lines on medium with tobramycin or gentamicin. The lines were produced with vectors pIT6 and pIT19, respectively (see Fig. 2). As a control, a transplastomic *Nt*-IT6 clone obtained by selection for spectinomycin resistance is also shown. **b** Additional regeneration rounds conducted in the presence of the selection agent to obtain homoplasmic transplastomic shoots. *Nt*-IT6 plates were photographed after 4 weeks, the *Nt*-IT19 plate after 6 weeks. **c** Rooting and growth of transplastomic lines under aseptic conditions. Tobramycin or gentamicin-resistant shoots from selection plates were grown on phytohormone-free medium in the presence of the antibiotic (30 mg/L tobramycin or gentamicin), the spectinomycin-resistant plant was grown in the presence of 500 mg/L spectinomycin

Introduction of the *aac6-aph2* gene as a passenger gene in transformation experiments with vector pIT6 followed by selection for spectinomycin resistance (Figs. 2b, 3) allowed us to determine the resistance levels of transplastomic lines to tobramycin, sisomicin and gentamicin. Based on regeneration tests on different concentrations of these three antibiotics and the previously performed antibiotic sensitivity tests of wild-type plants (Fig. S1), 30, 40 and 50 mg/L tobramycin, 25 and 35 mg/L sisomicin, and 50 mg/L gentamicin were chosen as concentrations for selection of transplastomic lines in initial plastid transformation experiments.

Selection for sisomicin resistance gave a number of regenerants (‘Primary resistant lines’; Table 1). However, additional regeneration rounds clearly showed that these putative lines were as sensitive to sisomicin as the wild type, whereas transplastomic *Nt*-IT6 controls lines that had been isolated by spectinomycin selection were resistant to 25 and 35 mg/L sisomicin. These results strongly suggested that all primary regenerants obtained by sisomicin selection are escapees rather than true transplastomic lines (Table 1).

In two independent transformation experiments, the bifunctional *aac6-aph2* resistance gene was combined with selection for gentamicin resistance. While one of the experiments produced only false positive lines, seven transplastomic lines were isolated from the second experiment and confirmed by both RFLP analysis and additional regeneration rounds on gentamicin-containing media (Fig. 3). However, the overall transformation efficiency obtained with gentamicin as selection agent was rather low and did not come close to the efficiency reached with the *aadA* and spectinomycin selection (Table 1).

By contrast, selection for tobramycin resistance yielded a large number of resistant lines, many of which were confirmed as true transplastomic events by their sustained resistance in subsequent regeneration rounds and by RFLP analyses (Figs. 2b, 3; Table 1). Transplastomic lines were recovered from all three antibiotic concentrations tested (30, 40 and 50 mg/L tobramycin). As expected, the number of false positive events (escapees) was higher on the low antibiotic concentrations (Table 1). Also, the vector with the strong expression signals driving the *aac6-aph2* selectable marker (pIT20) produced more transplastomic lines than the vector with the weaker expression signals (pIT19; Table 1).

To test if the background of false positive events can be eliminated by selecting for higher concentrations of tobramycin, transformation experiments with selection for 75 or 100 mg/L tobramycin were conducted. These experiments did not produce any resistant lines indicating that 30–50 mg/L is the suitable selection window for tobramycin (Table 1). To test whether the background regeneration can be attributed to the instability of tobramycin, the effect of a medium change was analyzed. To this end, the leaf pieces were transferred to fresh selection medium after 3–4 weeks of incubation. Indeed, this protocol substantially reduced background regeneration and increased selection efficiency (Table 1). In addition, the reexposure of primary regenerants to selection medium in a second regeneration round (Fig. 3) turned out to be a very efficient method of distinguishing escapees from true transformants, in that all candidate lines surviving this selection were transplastomic (Table 1).

Overall, the transformation efficiency with *aac6-aph2* as selectable marker in combination with tobramycin selection was similarly high as that with the standard marker gene *aadA* and spectinomycin selection (Table 1), thus providing an attractive alternative selection system for transplastomic plants.

### Characterization of transplastomic plants generated with *aac6-aph2* as selectable marker gene

**Fig. 4:**
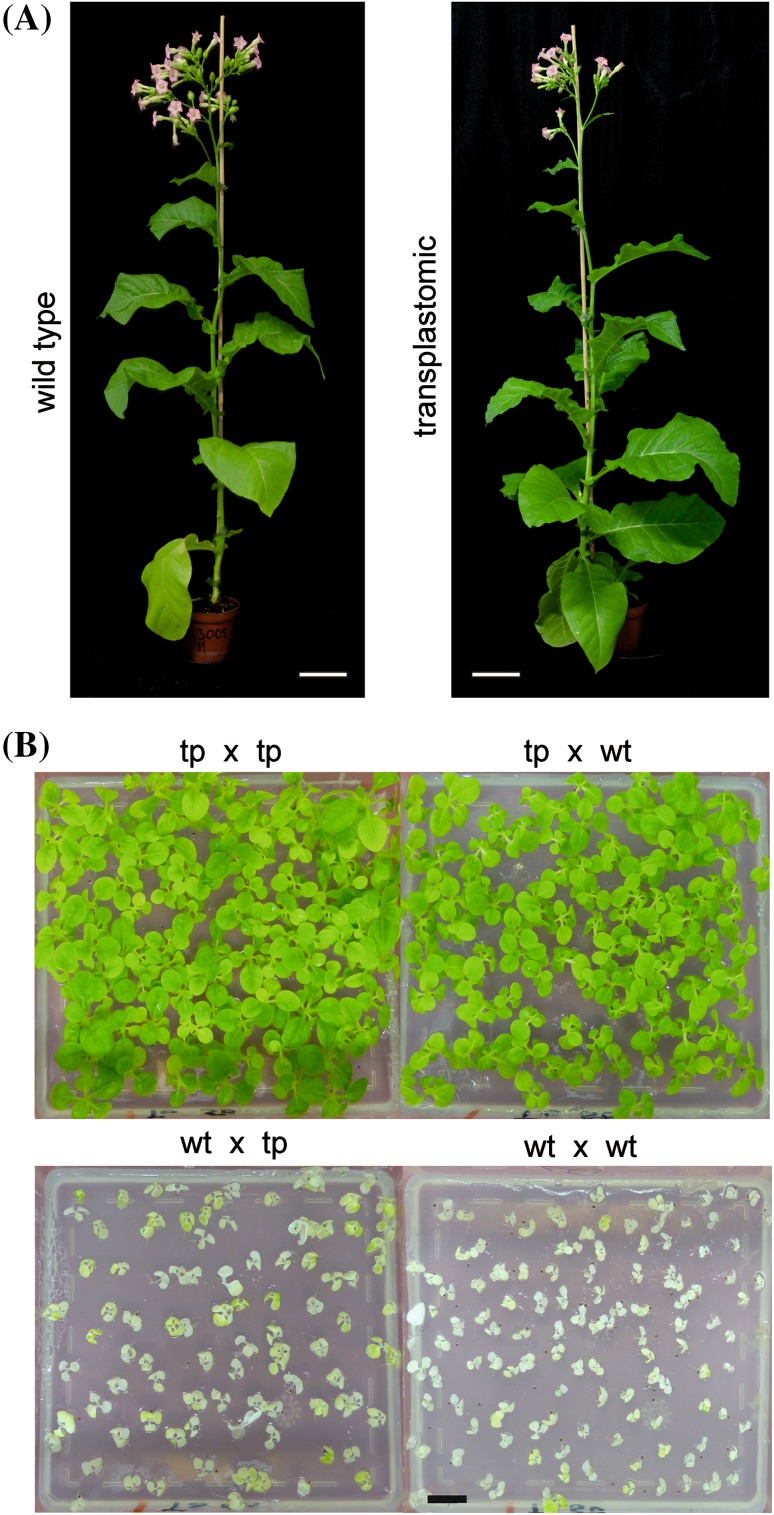
Growth of transplastomic plants to maturity and seed assays confirming maternal transgene inheritance. **a** Transplastomic plants with the inserted *aac6-aph2* marker gene (*right*) grow like wild-type plants (*left*) under greenhouse conditions. *Scale bars* 10 cm. **b** Seed assays to confirm stable maternal inheritance of the chloroplast-encoded tobramycin resistance gene. A transplastomic *Nt*-pIT6 plant (tp) was selfed (tp × tp) and reciprocally crossed to a wild-type plant (tp × wt, wt × tp). As a control for antibiotic sensitivity, the selfed wild type (wt × wt) was also included. While the progeny from all crosses with the transplastomic line as maternal parent are homogeneously resistant to tobramycin (50 mg/L), the progeny from crosses with a wild-type plant as maternal parent are uniformly sensitive to tobramycin. *Scale bar* 1 cm

Transplastomic line containing the *aac6-aph2* marker gene grew normally both under sterile conditions (Fig. 3c) and in the greenhouse (Fig. 4a). Plants grown in the greenhouse were phenotypically indistinguishable from wild-type plants (Fig. 4), were fertile and produced normal amounts of seeds.

To ultimately confirm homoplasmy of the transplastomic lines and to demonstrate maternal inheritance of the *aac6-aph2* marker, seed assays were conducted. To this end, reciprocal crosses between transplastomic plants and wild-type plants were conducted and the resulting seeds were germinated in the presence of tobramycin (Fig. 4b). As expected, the tobramycin resistance was stably transmitted into the next generation and displayed strictly maternal inheritance, as typical of plastid-encoded traits. Absence of tobramycin-sensitive seedlings from the progeny also ultimately verified homoplasmy of the transplastomic plants (Fig. 4b). Identical results were obtained when gentamicin resistance was tested by inheritance assays (Fig. S2).

Isolation of homoplasmic transplastomic lines with the established spectinomycin selection system based on the *aadA* marker gene typically requires two or three additional rounds of regeneration under antibiotic selection to eliminate residual copies of the wild-type plastid genome (Svab and Maliga 1993). When the primary transplastomic regenerants from selection for 50 mg/L tobramycin were analyzed by Southern blotting, we noticed that a high proportion of them was already homoplasmic (Fig. 2c; Table 1). This was not the case with the transplastomic lines isolated from selection for lower levels of tobramycin resistance (30 or 40 mg/L; Table 1) indicating that the strength of the selection pressure is responsible for this effect. Thus, although the primary selection of transplastomic lines with tobramycin takes, on average, longer than selection with spectinomycin (Table 1), considerable time is saved by faster attainment of homoplasmy.

### Cross-resistances to aminoglycoside antibiotics

**Fig. 5:**
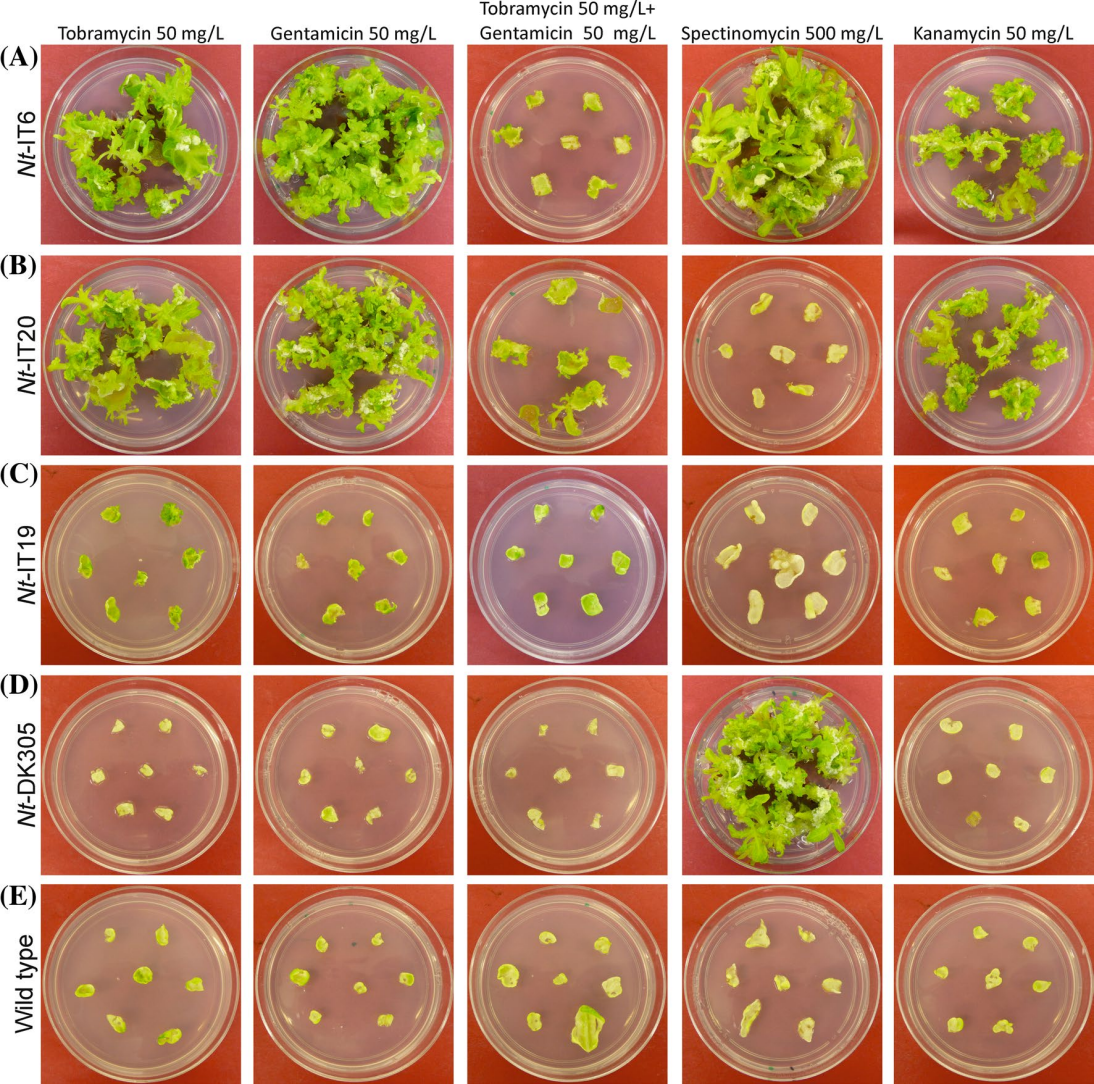
Specificity test of the *aadA* and *aac6-aph2* marker genes. The detoxification activity of the encoded enzymes towards the four aminoglycoside antibiotics tobramycin, gentamicin, spectinomycin and kanamycin was assayed by exposing leaf explants of transplastomic plants and a wild-type control to selective regeneration medium containing tobramycin, gentamicin, tobramycin + gentamicin, spectinomycin or kanamycin. Photographs were taken after 3 weeks. **a** An *Nt*-IT6 line harboring the *aac6-aph2* gene under the control of strong expression elements and additionally the *aadA* marker. **b** An *Nt*-IT20 line containing the *aac6-aph2* gene under the control of strong expression elements. **c** An *Nt*-IT19 line harboring the *aac6-aph2* gene under the control of weak expression elements. **d** An *Nt*-DK305 line expressing only the *aadA* marker gene. **e** A wild-type plant. Note that transplastomic lines containing only the *aadA* gene (*Nt*-DK305) cannot detoxify tobramycin and gentamicin. See also Fig. S3

The successful generation of transplastomic lines with the three different *aac6-aph2* vectors (Fig. 2) allowed us to determine the resistance levels to different aminoglycoside antibiotics and compare them to the wild type and to transplastomic plants generated with the standard selectable marker gene *aadA*. To this end, leaf explants of homoplasmic transplastomic plants were exposed to tobramycin, gentamicin, tobramycin + gentamicin, spectinomycin and kanamycin A. As expected, strong spectinomycin resistance was observed in *Nt*-DK305 and *Nt*-IT6 plants that harbor the *aadA* marker in their plastid genomes (Fig. 5). By contrast, *Nt*-IT19 and *Nt*-IT20 plants displayed no appreciable resistance to spectinomycin, demonstrating that the AAC6-APH2 enzyme cannot detoxify spectinomycin.

All transplastomic plants containing the *aac6-aph2* marker (*Nt*-IT6, *Nt*-IT19 and *Nt*-IT20) were capable of regenerating in the presence of tobramycin. However, regeneration was noticeably faster in plants that express the *aac6-aph2* gene from the strong ribosomal RNA operon promoter and the strongest known Shine-Dalgarno sequence (*G10L; Nt*-IT6 and *Nt*-IT20) than in plants where expression is driven by the considerably weaker heterologous *psbA* promoter and 5′ untranslated region from *Chlamydomonas* (*Nt*-IT19; Fig. 5; Fig. S3). This difference was even more pronounced when regeneration assays were performed in the presence of both tobramycin and gentamicin (50 mg/L each). This strong selection pressure substantially delayed regeneration in all transplastomic lines harboring the *aac6-aph2* transgene, but much more so in *Nt*-IT19 plants than in the *Nt*-IT6 and *Nt*-IT20 plants (Fig. 5; Fig. S3).

Finally, we also assayed all transplastomic lines for cross-resistance to kanamycin A. While the *aac6-aph2* gene did not confer increased tolerance to spectinomycin, it provided some resistance to kanamycin (Fig. 5). However, although kanamycin belongs to the 4,6-disubstituted aminoglycosides (like tobramycin and gentamicin) and was reported to be efficiently detoxified by the AAC6-APH2 enzyme in bacteria (Frase et al. 2012), the resistance level in all transplastomic *Nt*-IT lines was lower compared to tobramycin and gentamicin (Fig. 5), indicating that the *aac6-aph2* marker is not ideal for kanamycin selection of transplastomic plants.

## Discussion

In the course of this work, we have developed a novel selection marker for plastid transformation. The marker gene encodes a bifunctional enzyme that has both phosphotransferase and acetyltransferase activity (Fig. 1) towards a broad range of aminoglycoside antibiotics. We have shown that, when tethered to plastid expression signals, the *aac6-aph2* gene provides sufficiently strong resistance to tobramycin and gentamicin to select plastid transformants in tobacco. The homoplasmic transplastomic status of the selected lines was evidenced by RFLP analyses and inheritance assays (Figs. 2, 4; Table 1). Tobramycin selection appears to be superior to gentamicin selection (Table 1), and we, therefore, recommend to use the *aac6-aph2* marker in combination with selection for either 40 or 50 mg/L tobramycin.

The *aac6-aph2* marker provides an alternative selectable marker gene to the commonly used spectinomycin resistance gene *aadA*. The efficiency of both marker genes (number of transplastomic lines obtained per bombarded leaf sample) is very similar (Table 1). When comparing the standard *aadA*-based spectinomycin selection system with the *aac6-aph2*-based tobramycin selection, each system has potential advantages and disadvantages. The advantages of the *aac6-aph2* marker lie in the absence of spontaneous antibiotic-resistance mutants that frequently appear in spectinomycin selection for transplastomic lines (Svab et al. 1990; Svab and Maliga 1993, 1991; Bock 2001), and the faster attainment of homoplasmy in that a substantial number of primary transplastomic lines are already homoplasmic (Table 1). The latter may be attributable to the stronger selection pressure exerted by tobramycin selection compared to spectinomycin selection. This explanation is consistent with the narrower selection window for tobramycin, with tobramycin concentrations of 75 mg/L and above preventing selection of transplastomic cells (Table 1). Current disadvantages of the *aac6-aph2* marker lies in the somewhat longer primary selection phase (Table 1) which, however, is (over)compensated by the quicker attainment of homoplasmy, and the appearance of some escapees (i.e., regenerating plantlets that are not tobramycin resistant). Their frequency can be substantially reduced by a medium change during primary selection of transplastomic clones (Table 1). However, given the labor and cost involved in a complete medium change, it may be more practical to simply eliminate the escapees by an additional regeneration test on tobramycin-containing medium. The latter procedure reliably distinguished true transformants from escapees in that all lines that were resistant to tobramycin in an additional regeneration assay turned out to be true plastid transformants (Table 1).

New selection markers for chloroplast transformation are an important addition to our toolbox for plastid genome engineering for two reasons. First, they facilitate supertransformation of the plastid genome, that is the transformation of an already transplastomic plant with a second constructs, without the need for prior selectable marker recycling (Lutz and Maliga 2007; Day and Goldschmidt-Clermont 2011). Supertransformation is not only the method of choice for the successive introduction of multiple transgenes in multigene engineering efforts, it also facilitates the construction of multiple knock-outs or the site-directed mutagenesis of two unlinked genes in the plastid genome (Ehrnthaler et al. 2014). Secondly, new selectable marker genes will likely be instrumental in the extension of the plastid transformation technology to new species. The lack of efficient selection systems represents the major obstacle to the implementation of plastid transformation in monocot species, including cereals as the world’s most important food crops. Since most, if not all, cereal species are endogenously resistant to spectinomycin (Fromm et al. 1987), a breakthrough with plastid transformation in monocots will be critically dependent on the identification of selection agents that effectively inhibit callus growth in the dark (Ahmadabadi et al. 2007). Experiments are underway to assess the sensitivity of cereal cell cultures to tobramycin and gentamicin and test the *aac6-aph2* gene in combination with optimized expression signals for non-green plastids (Zhang et al. 2012; Caroca et al. 2013) for its suitability as a selectable marker for monocot plastid transformation. Preliminary experiments indicate that maize callus growth is sensitive to both tobramycin and gentamicin.

## Electronic supplementary material

Below is the link to the electronic supplementary material.

Supplementary material 1 (PDF 344 KB)
