# Supplementary material for: A bifunctional aminoglycoside acetyltransferase/phosphotransferase conferring tobramycin resistance provides an efficient selectable marker for plastid transformation
Source: Plant Mol Biol. 2016 Nov 17;93(3):269–81. doi: 10.1007/s11103-016-0560-x (PMC5306187; doi:10.1007/s11103-016-0560-x)
Supplement: Supplementary file 1 — Supplementary material 1 (PDF 344 KB) [file 11103_2016_560_MOESM1_ESM.pdf]

Supplementary Materials

Table S1. Sequences of coding regions and expression elements used in this study. SD: Shine-Dalgarno sequence.

**Element**	**Sequence (5' → 3')**
Nt Prrn (130 bp) *G10L*	CGGTACCCCAAAGCTCCCCCGCCGTCGTTCAATGAGAATGGATAAGAGGCTC<br>GTGGGATTGACGTGAGGGGGCAGGGATGGCTATATTTCTGGGAGCGAACTCC<br>GGGCGAATACGAAGCGCTTGGATACGGATCCAAATACTGCAGTTTAACTTTAA<br>GAAGGAGATATACCC
Nt T*rps16*	AGAAATTCAATTAAGGAAATAAATTAAGGAAATACAAAAAGGGGGGTAGTCATT<br>TGTATATAACTTTGTATGACTTTTCTCTTCTATTTTTTTGTATTTCCTCCCTTTCC<br>TTTTCTATTTGTATTTTTTTATCATTGCTTCCATT
Nt Prrn (175 bp) SD *rbcL*	*TAATTAATCGACAGATCCCGAATTGGGAT*CGGTACCCAAAGCTCCCCCGCCGT<br>CGTTCAATGAGAATGGATAAGAGGCTCGTGGGATTGACGTGAGGGGGCAGGG<br>ATGGCTATATTTCTGGGAGCGAACTCCGGGCGAATACGAAGCGCTTGGATACA<br>GTTGTAGGGAGGGATTT
Nt T*psbA*	TGTCTAGAGCGATCCTGGCCTAGTCTATAGGAGGTTTTGAAAAGAAAGGAGCA<br>ATAATCATTTTCTTGTTCTATCAAGAGGGTGCTATTGCTCCTTTCTTTTTTTCTTT<br>TTATTTATTTACTAGTATTTTACTTACATAGACTTTTTTGTTTACATTATAGAAAAA<br>GAAGGAGAGGTTATTTTCTTGCATTTATTCATGATTGAGTATTCTATTTTGATTTT<br>GTATTTGTTTAAAATTGTAGAAATAGAACTTGTTTCTCTTCTTGCTAATGTTACTA<br>TATCTTTTTGATTTTTTTTTTCCAAAAAAAAAATCAAATTTTGACTTCTTCTTATCT<br>CTTATCTTTGAATATCTCTTATCTTTGAAATAATAATATCATTGAAATAAGAAAGA<br>AGAGCTATATTCGACCT
Cr P*psbA*	GATCCAAGAAAAGTGAGCTATTAACGCGTCCTATTTTAATACTCCGAAGGAGG<br>CAGTTGGCAGGCAACTGCCACTGACGTCCCGTAAGGGTAAGGGGACGTCCAC<br>TGGCGTCCCGTAAGGGGAAGGGGACGTAGGTACATAAATGTGCTAGGTAACTA<br>ACGTTTGATTTTTTGTGGTATAATATATGTACCATGCTTTTAATAGAAGCTTGAA<br>TTTATAAATTAAAATATTTTTACAATATTTTACGGAGAAATTAAAACTTTAAAAAA<br>ATTAACAT
Cr T*rbcL*	AAGCTTGTACTCAAGCTCGTAACGAAGGTCGTGACCTTGCTCGTGAAGGTGGC<br>GACGTAATTCGTTCAGCTTGTAAATGGTCTCCAGAACTTGCTGCTGCATGTGAA<br>GTTTGGAAAGAAATTAAATTCGAATTTGATACTATTGACAAACTTTAATTTTTATT<br>TTTCATGATGTTTATGTGAATAGCATAAACATCGTTTTTATTTTTATGGTGTTTAG<br>GTTAAATACCTAAACATCATTTTACATTTTTAAAATTAAGTTCTAAAGTTATCTTT<br>TGTTTAAATTTGCCTGTCTTTATAAATTACGATGTGCCAGAAAAATAAAATCTTA<br>GCTTTTTATTATAGAATTTATCTTTATGTATTATATTTTATAAGTTATAATAAAAGA<br>AATAGTAACATACTAAAGCGGATGTAACTCAATCGGTAGAGTGCGATCC

aac6-aph2 gene	ATGGAAAATATTGTAGAAAATGAAATTTGTATTCGAACTTTAATTGATGATGATT<br>TTCCTTTAATGTTAAAATGGTTAACTGATGAAAGAGTTTTAGAATTTTATGGAGG<br>TAGAGATAAAAAATATACTTTAGAATCTTTAAAAAAACATTATACTGAACCTTGG<br>GAAGATGAAGTATTTAGAGTAATTATTGAATATAATAATGTTCCTATTGGTTATG<br>GACAAATTTATAAAATGTATGATGAATTATATACTGATTATCATTATCCTAAAACT<br>GATGAAATTGTATATGGAATGGATCAATTTATTGGTGAACCTAATTATTGGTCTA<br>AAGGTATTGGTACTCGATATATTAAATTAATTTTTGAATTTTTAAAAAAAGAACG<br>AAATGCTAATGCTGTAATTTTAGATCCTCATAAAAATAATCCACGAGCTATTCGA<br>GCTTATCAAAAATCTGGATTTAGAATTATTGAAGATTTACCTGAACATGAATTAC<br>ATGAAGGAAAAAAAGAAGATTGTTATTTAATGGAATATAGATATGATGATAATGC<br>TACTAATGTTAAAGCTATGAAATATTTAATTGAACATTATTTTGATAATTTTAAAG<br>TAGATTCTATTGAAATTATTGGTTCTGGATATGATTCTGTTGCTTATTTAGTTAAT<br>AATGAATATATTTTTAAAACTAAATTTTCTACTAATAAAAAAAAAGGTTATGCTAA<br>AGAAAAAGCTATTTATAATTTTTTAAATACTAATTTAGAAACTAATGTAAAAATTC<br>CTAATATTGAATATTCTTATATTTCTGATGAATTATCTATTTTAGGATATAAAGAA<br>ATTAAAGGTACTTTTTTAACTCCTGAAATTTATTCTACTATGTCTGAAGAAGAAC<br>AAAATTTATTAAAACGAGATATTGCTTCTTTTTTAAGACAAATGCATGGTTTAGA<br>TTATACTGATATTTCTGAATGTACTATTGATAATAAACAAAATGTATTAGAAGAAT<br>ATATTTTATTACGAGAAACTATTTATAATGATTTAACTGATATTGAAAAAGATTAT<br>ATTGAATCTTTTATGGAAAGATTAAATGCTACTACTGTTTTTGAAGGTAAAAAAT<br>GTTTATGTCATAATGATTTTTCTTGTAATCATTTATTATTAGATGGAAATAATAGA<br>TTAACTGGAATTATTGATTTTGGTGATTCTGGAATTATTGATGAATATTGTGATT<br>TTATTTATTTATTAGAAGATTCTGAAGAAGAAATTGGTACTAATTTTGGAGAAGA<br>TATTTTACGAATGTATGGAAATATTGATATTGAAAAAGCTAAAGAATATCAAGAT<br>ATTGTAGAAGAATATTATCCTATTGAAACTATTGTATATGGAATTAAAAATATTAA<br>ACAAGAATTTATTGAAAATGGTCGAAAAGAAATTTATAAAAGAACTTATAAAGAT<br>TAA
aadA gene	ATGGCAGAAGCGGTGATCGCCGAAGTATCGACTCAACTATCAGAGGTAGTTGG<br>CGTCATCGAGCGCCATCTCGAACCGACGTTGCTGGCCGTACATTTGTACGGCT<br>CCGCAGTGGATGGCGGCCTGAAGCCACACAGTGATATTGATTTGCTGGTTACG<br>GTGACCGTAAGGCTTGATGAAACAACGCGGCGAGCTTTGATCAACGACCTTTT<br>GGAAACTTCGGCTTCCCCTGGAGAGAGCGAGATTCTCCGCGCTGTAGAAGTC<br>ACCATTGTTGTGCACGACGACATCATTCCGTGGCGTTATCCAGCTAAGCGCGA<br>ACTGCAATTTGGAGAATGGCAGCGCAATGACATTCTTGCAGGTATCTTCGAGC<br>CAGCCACGATCGACATTGATCTGGCTATCTTGCTGACAAAAGCAAGAGAACAT<br>AGCGTTGCCTTGGTAGGTCCAGCGGCGGAGGAACTCTTTGATCCGGTTCTTGA<br>ACAGGATCTATTTGAGGCGCTAAATGAAACCTTAACGCTATGGAACTCGCCGC<br>CCGACTGGGCTGGCGATGAGCGAAATGTAGTGCTTACGTTGTCCCGCATTTGG<br>TACAGCGCAGTAACCGGCAAAATCGCGCCGAAGGATGTCGCTGCCGACTGGG<br>CAATGGAGCGCCTGCCGGCCCAGTATCAGCCCGTCATACTTGAAGCTAGACA<br>GGCTTATCTTGGACAAGAAGAAGATCGCTTGGCCTCGCGCGCAGATCAGTTGG<br>AAGAATTTGTCCACTACGTGAAAGGCGAGATCACCAAGGTAGTCGGCAAATAA

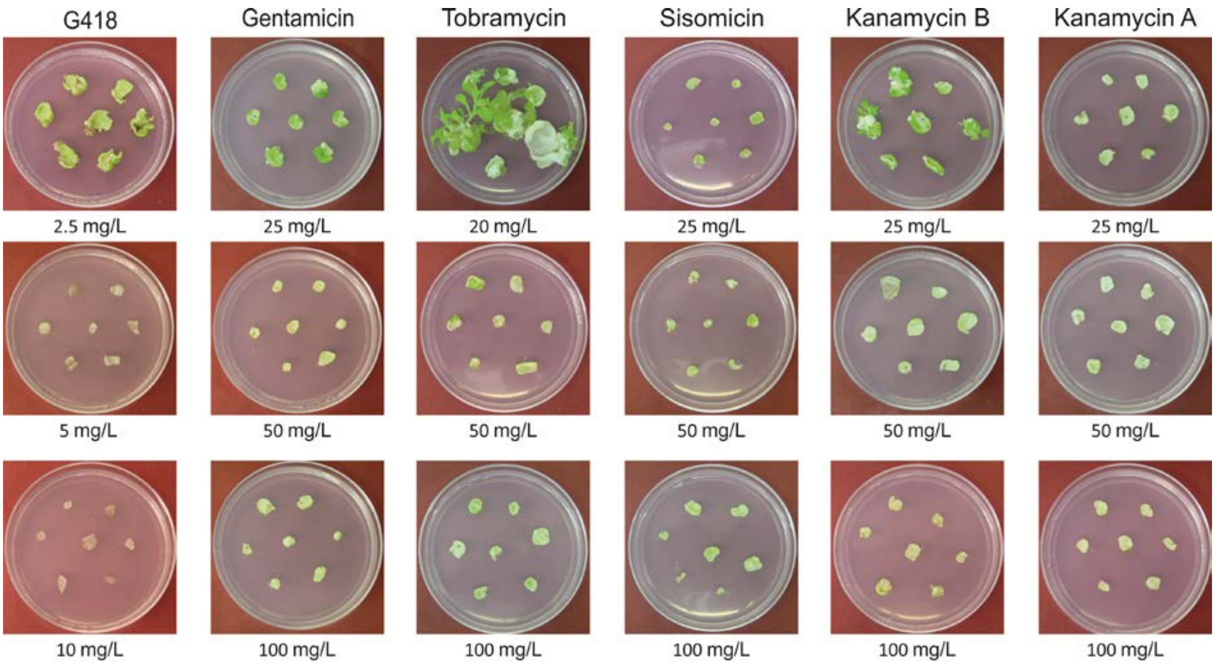

Figure S1. Antibiotic sensitivity assays to determine the effective selection windows for the aminoglycoside antibiotics tested in this study. Leaf pieces of *Nicotiana tabacum* wild-type plants were exposed to regeneration medium supplemented with different concentrations of six different aminoglycosides and photographed after 8 weeks. Tobramycin (30, 40, 50, 75 and 100 mg/L), sisomicin (25 and 35 mg/L) and gentamicin (50 mg/L) were chosen as selection agents in plastid transformation experiments.

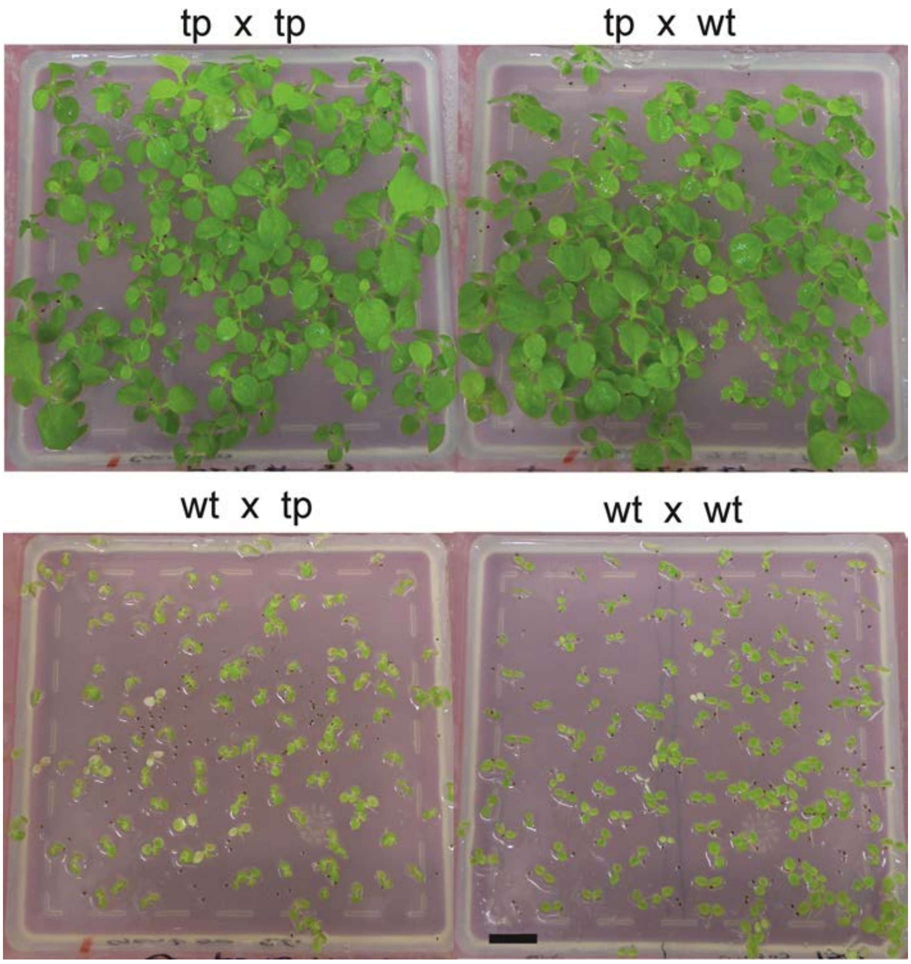

Figure S2. Seed assays on gentamicin-containing medium to confirm stable maternal inheritance of the chloroplast-encoded bifunctional resistance gene. A transplastomic *Nt*-pIT6 plant (tp) was selfed (tp×tp) and reciprocally crossed to a wild-type plant (tp×wt, wt×tp). As a control for antibiotic sensitivity, the selfed wild type (wt×wt) was also included. The progeny from all crosses with the transplastomic line as maternal parent are homogeneously resistant to gentamicin (50 mg/L). By contrast, the progeny from crosses with a wild-type plant as maternal parent are uniformly sensitive to the antibiotic. Scale bar: 1 cm.

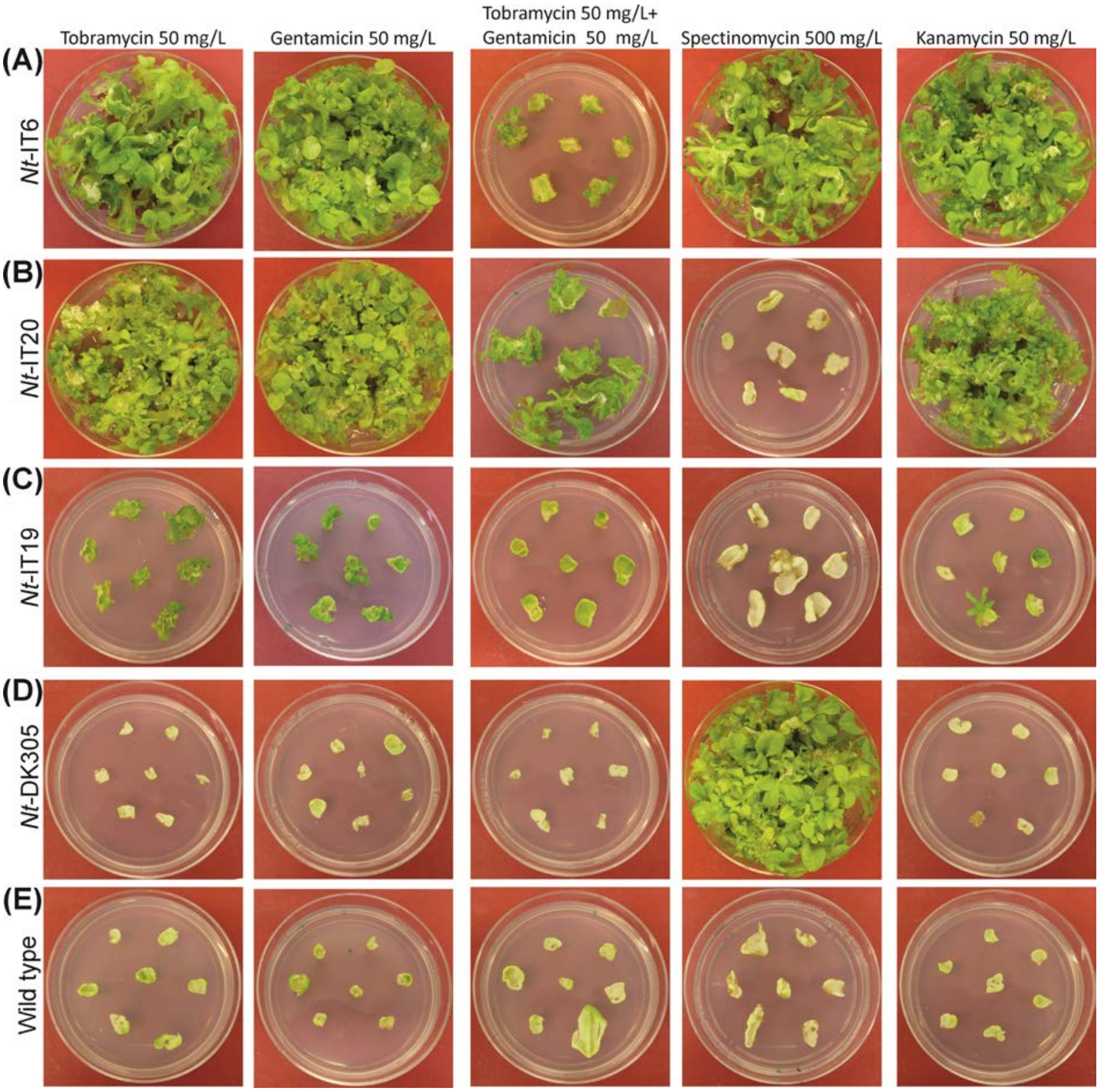

Figure S3. Specificity test of the *aadA* and *aac6-aph2* marker genes. The assay shown in Fig. 5 was photographed two weeks later (i.e., after 5 weeks of incubation). (**A**) An *Nt*-IT6 line harboring the *aac6-aph2* gene under the control of strong expression elements and additionally the *aadA* marker. (**B**) An *Nt*-IT20 line containing the *aac6-aph2* gene under the control of strong expression elements. (**C**) An *Nt*-IT19 line harboring the *aac6-aph2* gene under the control of weak expression elements. (**D**) An *Nt*-DK305 line expressing only the *aadA* marker gene. (**E**) A wild-type plant. Note incipient regeneration from the *Nt*-IT19 explants after 5 weeks (cf. Fig. 5).
